# Evaluation of Alginate‐Gum Arabic Coating and Pomegranate (*Punica granatum* L.) Peel Extract Impact on Microbial and Physicochemical Properties of Shrimp (*Litopenaeus vannamei*) During Refrigerated Storage

**DOI:** 10.1002/fsn3.71340

**Published:** 2025-12-21

**Authors:** Fatemeh Rahmati, Sara Jafarian, Peiman Ariaii, Mahdi Sharifi Soltani, Leila Roozbeh Nasiraei

**Affiliations:** ^1^ Department of Food Science and Technology Islamic Azad University Nour Iran; ^2^ Department of Food Science and Technology Islamic Azad University Amol Iran; ^3^ Department of Veterinary, Agriculture Faculty Islamic Azad University Chalous Iran; ^4^ Department of Veterinary, Microbiology Faculty Islamic Azad University Nour Iran

**Keywords:** antimicrobial activity, antioxidant properties, edible coating, *Litopenaeus vannamei*, natural preservative, sensory attributes, shelf life extension

## Abstract

This study evaluated the impact of alginate‐gum Arabic coating and pomegranate ( *Punica granatum* L.) peel extract (PPE) on the microbial and physicochemical properties of shrimp ( *Litopenaeus vannamei* ) during refrigerated storage. The treatments included T1 (0.5% encapsulated PPE), T2 (1% encapsulated PPE), T3 (1% free‐form PPE), T4 (2% free‐form PPE), T5 (1% alginate‐gum Arabic coating), and T6 (control). Results indicated that the peroxide value ranged from 0.42 meq/kg (T2, day 3) to 2.15 meq/kg (T6, day 15); TBARS was lowest at 0.18 mg MDA/kg (T2, day 3) and highest at 1.02 mg MDA/kg (T5, day 9); free fatty acid (FFA) varied from 0.35% (T1, day 3) to 1.85% (T6, day 15); pH ranged from 7.34 (T1, day 3) to 8.30 (T5, day 9). Firmness peaked at 4.5 N (T2, day 3) and was lowest at 2.1 N (T6, day 15); resilience was highest at 0.85 (T1, day 3) and lowest at 0.45 (T6, day 15); cohesiveness and adhesiveness showed no significant differences (*p* > 0.05), with values slightly higher on day 15 (cohesiveness: 0.75–0.82; adhesiveness: −1.2 to −0.9 g/s). Sensory scores for taste, texture, color, odor, and overall acceptance were highest in T1 and T2 (4.5–4.8 on day 3, declining to 3.2–3.5 on day 15). Total bacterial count (TBC) was lowest at 2.5 log CFU/g (T2, day 3) and highest at 6.8 log CFU/g (T6, day 15). The highest saturated fatty acid (SFA), monounsaturated fatty acid (MUFA), and polyunsaturated fatty acid (PUFA) were palmitic acid (20.91%), elaidic acid (26.91%), and linoleic acid (7.53%), respectively, on day 3, with better preservation in T1 and T2. Overall, 0.5% (T1) and 1% (T2) encapsulated PPE incorporation provided the best physicochemical and sensory properties compared to other treatments. PPE is a viable natural preservative for maintaining the quality of shrimp and other seafood products during refrigerated storage.

## Graphical abstract

This study aimed to evaluate the impact of alginate‐gum arabic and pomegranate (*Punica granutum* L.) peel extract on the microbial and physiochemical properties of shrimp (*Litopenaeus vannamei*) samples during refrigerator storage. In this research, we concluded that applying pomegranate peel extracts in shrimp samples is a viable strategy to preserve the physiochemical and microbiological quality of shrimp and other seafood products.

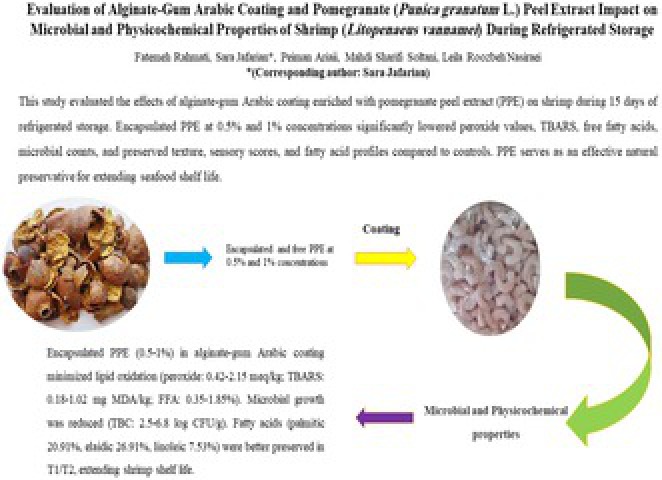

## 1 Introduction

Fresh seafood products have great nutritional value, including high concentrations of omega‐3 fatty acids, proteins, and vitamins, making them vital dietary components. However, seafood, particularly shrimp, is highly perishable and susceptible to chemical and microbial deterioration during storage (İlknur and Elsheikh 2023). Alginate, a polysaccharide derived from brown algae, and gum Arabic, a natural exudate from Acacia trees, are commonly used in edible coatings due to their film‐forming properties, biocompatibility, and ability to act as barriers against oxygen and moisture. Pomegranate ( *Punica granatum* L.) peel extract (PPE) is rich in bioactive compounds such as polyphenols, tannins, and flavonoids, which exhibit strong antimicrobial and antioxidant activities. The combination of alginate‐gum Arabic coating with PPE aims to create a synergistic edible film that extends shelf life by inhibiting microbial growth and oxidative rancidity in shrimp ( *Litopenaeus vannamei* ) during refrigerated storage. Cooling or freezing alone cannot fully inhibit lipid oxidation, protein denaturation, or microbiological activity. Consequently, the integration of processing and packaging technologies, such as edible coatings, has gained considerable traction (Dehghani et al. 2018). Shrimp ( *Litopenaeus vannamei* ) is abundant in the Iranian market and prized for its high protein and mineral content. However, its susceptibility to spoilage demands effective preservation strategies, including coatings, chilling, and freezing to retard bacterial proliferation and preserve quality (Mehraie et al. 2023).

Edible biopolymer coatings have emerged as a promising approach for prolonging the shelf life of food products (Salama et al. 2019; El‐Kholy et al. 2022). Formulated from polysaccharides, proteins, lipids, or their composites, these coatings provide biodegradability, recyclability, and sustainability (Moradinezhad et al. 2025). The incorporation of antioxidants and antimicrobial agents, such as essential oils, into edible coatings can further augment food safety and extend shelf life (Muñoz‐Tebar et al. 2023; Kumar et al. 2022). Microencapsulation safeguards sensitive compounds (e.g., polyphenols and volatile additives) against environmental stressors, ensuring stability and controlled release (Rashid et al. 2022; Calderón‐Oliver and Ponce‐Alquicira 2022).

Pomegranate ( *Punica granatum* L.), indigenous to the Middle East, is replete with bioactive compounds, particularly in its peel, which exhibits potent antimicrobial, antiviral, and antioxidant properties (Kalaycıoğlu and Erim 2017; Giri et al. 2023). Pomegranate peel harbors elevated levels of polyphenols, rendering it a robust natural preservative (Senturk Parreidt et al. 2018; Akhtar et al. 2015). Gum arabic and alginate, extensively employed in edible coatings, confer superior film‐forming and encapsulating attributes, thereby enhancing the preservation of perishable foods (El‐Kholy et al. 2022; Sood and Gupta 2015).

Previous studies on edible coatings for seafood preservation have primarily focused on single biopolymers or synthetic additives, often overlooking the synergistic effects of combined natural extracts like PPE with composite coatings such as alginate‐gum Arabic (e.g., limitations in controlled release and long‐term stability noted in studies using gelatin or chitosan alone; 102. Berizi et al. 2016; Hosseini et al. 2016). For instance, while gelatin‐based coatings with propolis have shown efficacy in fish fillets, they lack the specific polyphenol profile of PPE for targeted antioxidant action (Nessianpour et al. 2019). Similarly, chitosan‐PPE films have demonstrated antimicrobial properties but were limited in moisture barrier efficiency compared to alginate‐gum Arabic composites (Yuan et al. 2016; Nair et al. 2020). These gaps highlight the need for innovative composite coatings that integrate encapsulated PPE to address both microbial and oxidative spoilage more effectively. The current study fills this void by investigating the novel encapsulation of PPE within alginate‐gum Arabic coatings, providing enhanced controlled release and bioavailability. This approach not only extends shrimp shelf life but also offers environmental benefits through utilization of agricultural waste (pomegranate peels), reduces reliance on synthetic preservatives, and improves nutritional retention, potentially benefiting the seafood industry in terms of sustainability and consumer health.

This study aimed to evaluate the effects of an alginate‐gum arabic coating enriched with nanoparticle‐encapsulated pomegranate peel extract (PPE) on the microbial, physicochemical, and sensory attributes of shrimp (*Litopenaeus vannamei*) during refrigerated storage at 4°C for 15 days. The hypothesis posited that PPE, especially in its encapsulated form, would augment the shelf life and quality of shrimp relative to controls and alternative treatments. Notably, this investigation introduces a novel combination of alginate and gum arabic as a biopolymer matrix for nanoparticle‐encapsulated PPE, addressing gaps in seafood‐specific applications where prior studies have predominantly utilized chitosan‐based systems or non‐encapsulated extracts (Ebadi et al. 2019; Khodanazary 2019; Khodanazary et al. 2019; Rahul et al. 2024). This approach leverages the synergistic film‐forming properties of alginate‐gum arabic blends to enhance encapsulation efficiency and controlled release of PPE's bioactive polyphenols, potentially offering superior antimicrobial efficacy and oxidative stability compared to conventional coatings, while minimizing sensory alterations in delicate seafood products.

## 2 Materials and Methods

Preparation of Pomegranate Peel Extract (PPE) Pomegranate fruits ( *Punica granatum* L.) were purchased from a local market, washed with distilled water, peeled, and separated into edible and non‐edible sections. The peels were air‐dried at 40°C and 32% ± 4% relative humidity for 48 h until reaching a constant weight (moisture content: 13.3%). Dried peels were stored at −70°C, milled into a fine powder using an electric blender (Philips, HR 2815, Japan), and passed through a 470‐μm sieve. The powder (100 g) was suspended in 800 mL of double‐distilled water at 40°C, shaken for 24 h at 150 rpm on an orbital shaker (Model OS‐200, Optima, Japan), and filtered through Whatman No. 2 filter paper to remove particles, as described by Kharchoufi et al. (2018). The extract was concentrated under vacuum at 40°C using a rotary evaporator (Heidolph, Laborota 4000, Germany) to achieve a final concentration of 10% *w*/*v* and stored at −20°C until use.

Preparation of Encapsulated PPE For encapsulation, PPE was microencapsulated using spray drying with maltodextrin as the carrier material at a core‐to‐wall ratio of 1:3. The mixture was homogenized at 10,000 rpm for 5 min using an Ultra‐Turrax homogenizer (IKA T25, Germany), then spray‐dried (Buchi B‐290, Switzerland) with inlet air temperature of 150°C, outlet 80°C, and feed flow rate of 5 mL/min. The encapsulated powder was collected and stored in airtight containers at 4°C (modified from Calderón‐Oliver and Ponce‐Alquicira 2022).

Preparation of Alginate‐Gum Arabic Coating Solution Sodium alginate (1% *w*/*v*, Sigma‐Aldrich, USA) and gum Arabic (1% *w*/*v*, Merck, Germany) were dissolved separately in distilled water at 50°C with constant stirring for 30 min. The solutions were combined in a 1:1 ratio; glycerol (0.5% *v*/*v*, as plasticizer) was added, and the mixture was homogenized at 5000 rpm for 10 min. For treatments with PPE, encapsulated or free‐form PPE was incorporated at specified concentrations and stirred for an additional 15 min to ensure uniform distribution.

Treatments Six treatments were applied to shrimp samples: T1 (0.5% encapsulated PPE), T2 (1% encapsulated PPE), T3 (1% free‐form PPE), T4 (2% free‐form PPE), T5 (1% alginate‐gum Arabic coating without PPE), and T6 (control, no coating or PPE) (Table 1). Fresh shrimp (*Litopenaeus vannamei*, average weight 15–20 g each) were obtained from a local aquaculture farm, transported on ice, and processed within 2 h.

**TABLE 1:** Shrimp treatments with alginate‐gum arabic and pomegranate peel extract.

T1	T2	T3	T4	T5	T6
0.5% concentration of the PPE capsulate form	1% concentration of the PPE capsulate form	1% concentration of the PPE‐free form	2% concentration of the PPE‐free form	Coated with gum concentration of 1%	Control treatment

**FIGURE 1:**
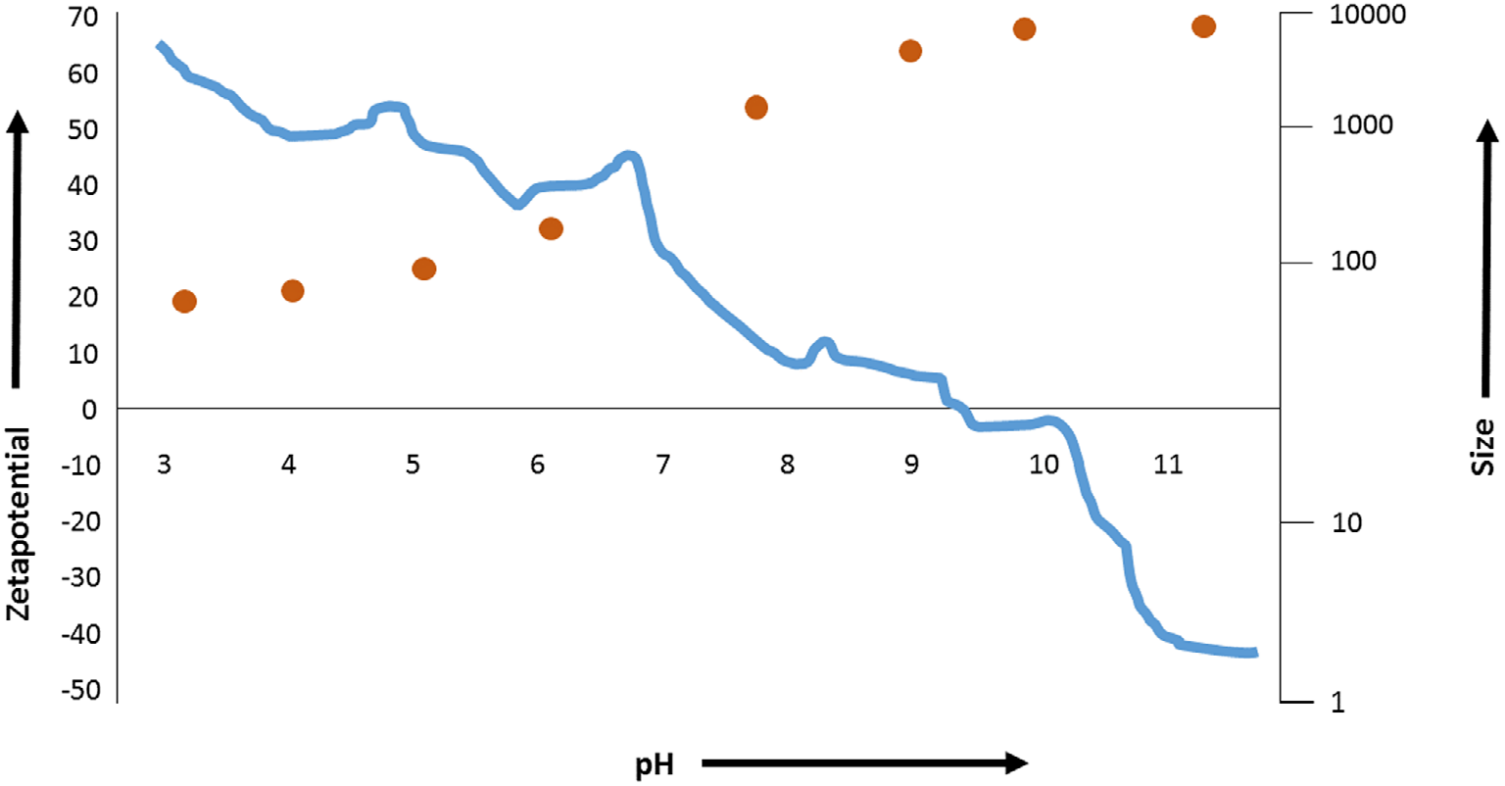
Zeta potential of pomegranate ( *Punica granatum* L.) peel extract.

**FIGURE 2:**
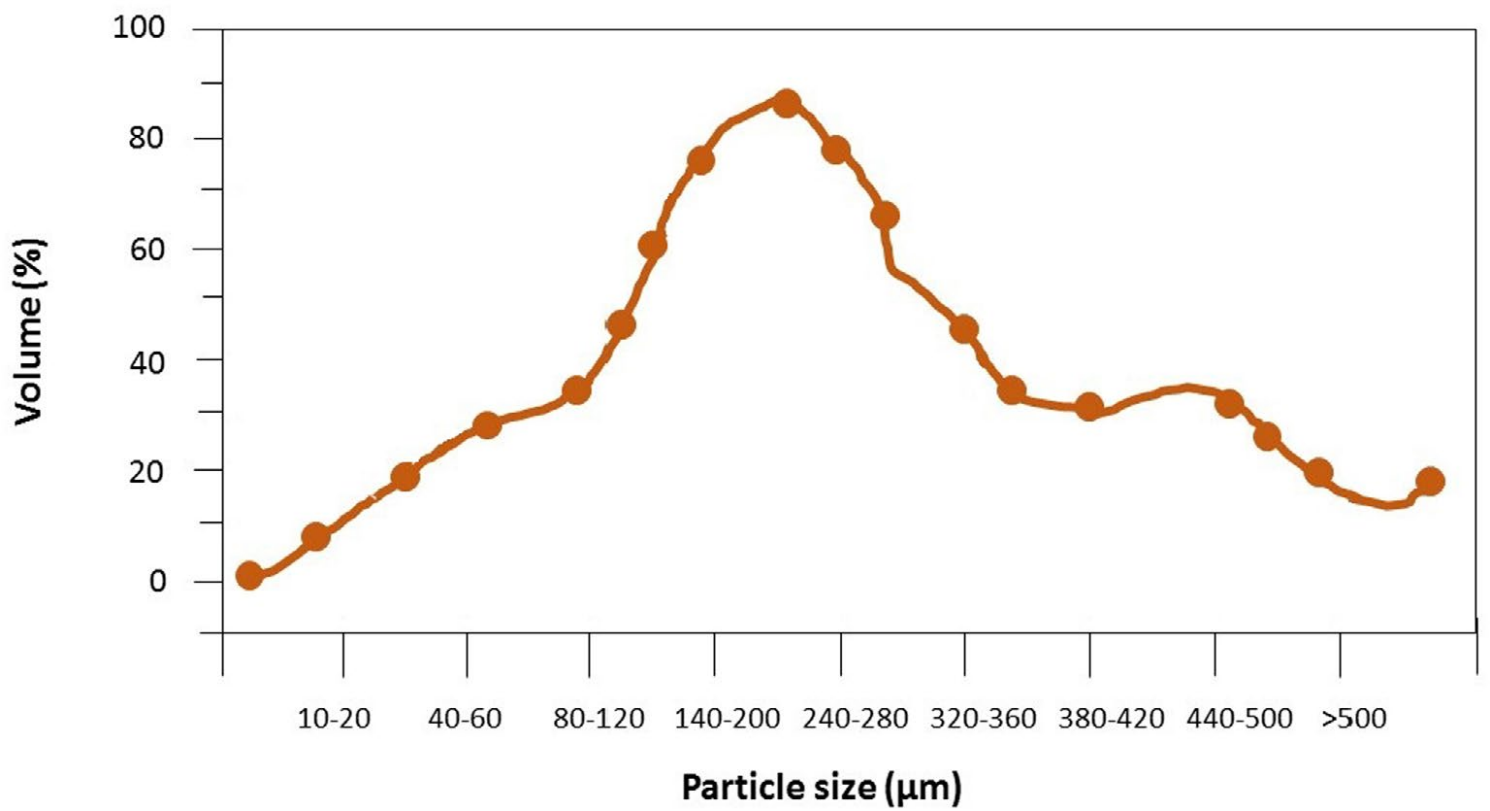
Particle size of pomegranate ( *Punica granatum* L.) peel extract.

The particle size distribution of microencapsulated pomegranate peel extract (PPE), measured at 25°C and pH 7, reveals a heterogeneous population of particles with diameters spanning various ranges (Table 2). The zeta potential of pomegranate ( *Punica granatum* L.) peel extract serves as a key indicator of colloidal stability for nanoparticles (Figure 1). Figure 2 showed the particle size of pomegranate ( *Punica granatum* L.) peel extract, determined via dynamic light scattering (DLS).

**TABLE 2:** Particle size distribution values of microencapsulated pomegranate peel extract (PPE) (at 25°C and pH 7).

Treatment	Particle size (μm)				Span number
	D59 right	D59 left	D88	D3	
PPE	78.2 ± 67.25	332.8 ± 33.95	233.4 ± 66.03	3.0 ± 93.09	265.48 ± 20.54

Samples were washed with chilled distilled water (4°C), drained, and dipped in the respective coating solutions for 1 min, followed by draining for 30 s and air‐drying at 4°C for 2 h to form the film. Treated samples (100 g per package) were placed in sterile polyethylene bags, sealed, and stored at 4°C ± 1°C for 15 days. Analyses were performed on days 0, 3, 6, 9, 12, and 15, with three replicates per treatment.

HPLC Analysis High‐performance liquid chromatography (HPLC, Knauer, Germany) with a Pump K‐1000 and UV‐visible detector was used, equipped with a C18‐SEC column (250 mm length, 4.8 mm i.d.). For injection, 1 g of PPE was dissolved in 10 mL distilled water, centrifuged at 4500 rpm for 4 min, and filtered through a 0.45‐μm filter. A 20 μL sample was injected at 25°C, using acetonitrile/distilled water/acetic acid as the mobile phase at a flow rate of 0.5 mL/min. Phenolic compounds were detected at 270 nm using standard solutions of gallic acid, punicalagin, and ellagic acid (Li et al. 2015; Karami et al. 2019).

Total phenolic content was determined using the Folin–Ciocalteu method, expressed as gallic acid equivalents (mg GAE/g) (Kennas et al. 2020). Specifically, 0.5 mL of diluted PPE was mixed with 2.5 mL of 10% Folin–Ciocalteu reagent and 2 mL of 7.5% sodium carbonate, incubated at room temperature for 30 min, and absorbance measured at 765 nm using a spectrophotometer (UV‐1800, Shimadzu, Japan). A calibration curve of gallic acid (0–100 mg/L) was used for quantification.

DPPH Radical Scavenging Activity The DPPH assay was conducted as described by Ahmed et al. (2015) with modifications. A 2.5 mL DPPH working solution was mixed with 500 μL diluted PPE, incubated in the dark for 30 min, and absorbance was measured at 517 nm. Radical scavenging activity (RSA) was calculated as follows: RSA (%) = ([*A*_c_—*A*_s_]/*A*_c_) × 100, where *A*_c_ and *A*_s_ are the absorbance of the control (500 μL methanol) and sample, respectively. The DPPH solution was prepared fresh at 0.1 mM in methanol, and measurements were taken using a spectrophotometer (UV‐1800, Shimadzu, Japan).

FRAP Radical Scavenging Assay The FRAP reagent, consisting of acetate buffer (300 mM, pH 3.6), TPTZ (10 mM in 40 mM HCl), and FeCl_3_·6H_2_O (20 mM) in a 10:1:1 ratio, was mixed with 500 μL diluted PPE. After 6 min of incubation, absorbance was measured at 593 nm, with results expressed using a FeSO_4_ standard curve (Li et al. 2006). The reaction was performed at 37°C, and absorbance was read using a spectrophotometer (UV‐1800, Shimadzu, Japan).

Peroxide value was determined by dissolving 20 g of shrimp sample in 30 mL *n*‐hexane to extract oil. A 5 g oil sample was mixed with glacial acetic acid and chloroform (3:2 ratio), followed by 0.5 mL saturated KI. After incubation in the dark, 30 mL distilled water was added, and titration was performed with 0.1 N sodium thiosulfate using starch as an indicator. Peroxide value was calculated as follows: Peroxide Value (meq/kg) = ([*V*—*V*_b_] × *N* × 1000)/*W*, where *V* and *V*_b_ are the volumes of sodium thiosulfate for the sample and blank (mL), *N* is the normality of sodium thiosulfate, and *W* is the oil weight (g) (Khalili et al. 2020). Titration was conducted until the blue color disappeared, and blanks were run in parallel.

TBARS Thiobarbituric acid reactive substances (TBARS) were measured using a modified method (Kamkar et al. 2010). TBA reagent (15 g trichloroacetic acid, 0.375 g thiobarbituric acid, 2 mL HCl, 82.9 mL distilled water) was mixed with the oil sample, heated at 95°C–100°C for 30 min, cooled, centrifuged, and absorbance measured at 532 nm. Results were expressed as mg MDA/kg sample using a standard curve of 1, 1, 3, 3‐tetraethoxypropane. Samples (10 g) were homogenized with 50 mL distilled water before adding 20 mL TBA reagent, and centrifugation was at 3000 rpm for 10 min (Sigma 3‐18K, Germany).

Free Fatty Acid (FFA) was measured as per Kalem et al. (2018). A 25 g shrimp sample was dissolved in 137 mL chloroform with anhydrous sodium sulfate, filtered, and mixed with five drops of 1% ethanolic phenolphthalein. Titration was performed with 1 N KOH until a pink color appeared. FFA was calculated as follows: FFA (%) = (*A* × *N* × 28.2)/*W*, where *A* is the volume of KOH (mL), *N* is the normality, and *W* is the sample weight (g). The mixture was shaken vigorously during titration to ensure complete reaction.

pH Index A 5 g shrimp sample was homogenized in 20 mL distilled water using a blender (Waring Commercial Blendor, USA) for 5 min, and pH was measured using a standardized pH meter (Nardoia et al. 2018). The pH meter (Metrohm 827, Switzerland) was calibrated with buffers at pH 4.0 and 7.0 before each measurement.

Sensory Evaluation Shrimp samples were cooked in boiling water for 3 min and evaluated by 50 trained panelists (25 men, 25 women, aged 20–40 years) using a 5‐point hedonic scale (5 = like significantly, 1 = dislike). Attributes assessed included taste, texture, color, odor, and overall acceptance. Informed consent was obtained from all panelists prior to evaluation. Panelists were trained in two sessions to familiarize them with the scale and attributes, and evaluations were conducted in individual booths under white light.

Microbial Analysis Total bacterial count (TBC) was determined by homogenizing 10 g shrimp in 90 mL sterile peptone water (0.1%), followed by serial dilutions and plating on plate count agar (PCA, Merck, Germany). Plates were incubated at 30°C for 48 h, and colonies were counted as log CFU/g (ISO 4833‐1:2013). Psychrotrophic bacteria were enumerated similarly but incubated at 7°C for 10 days.

Fatty acid profile fatty acids were extracted using chloroform‐methanol (2:1 *v*/*v*), methylated with boron trifluoride‐methanol, and analyzed by gas chromatography (Agilent 6890, USA) with a flame ionization detector and HP‐88 column (100 m × 0.25 mm). Carrier gas was helium at 1 mL/min, with oven temperature programmed from 140°C to 240°C at 4°C/min. Identification was based on retention times of standards (Supelco 37 FAME mix).

Statistical Analysis Data were analyzed using SPSS 22 software, with means compared using Tukey's test at a 95% confidence level (*p* < 0.05). Graphs were created using Excel.

## 3 Results and Discussion

The proximate composition of pomegranate peel extract (PPE) is detailed in Table 3, revealing total organic matter (TOM) as the predominant component at 97.10% ± 2.84%, while ash content was the lowest at 3.44% ± 0.10%. Other constituents included moisture (9.90% ± 0.29%), protein (6.55% ± 0.19%), crude fiber (10.55% ± 0.30%), lipid (3.47% ± 0.10%), nitrogen‐free extract (NFE) (76.50% ± 2.24%), neutral detergent fiber (NDF) (29.52% ± 0.86%), acid detergent fiber (ADF) (19.32% ± 0.56%), and acid detergent lignin (ADL) (3.92% ± 0.11%). These results indicate a high organic content, primarily driven by carbohydrates and fiber, with relatively low ash and lipid levels, suggesting PPE's potential as a nutrient‐rich additive for food preservation. These findings are consistent with Wanderley et al. (2023), who reported comparable values for pomegranate peel, including lipid (0.98%), protein (3.40%), fiber (11.00%), and ash (1.03%). Similarly, Basharat et al. (2023) documented moisture (7.28% ± 1.50%), crude fiber (13.91% ± 0.02%), fat (1.61% ± 0.08%), protein (3.78% ± 0.14%), and ash (3.63% ± 0.05%), reinforcing the reliability of the current data. Variations in composition may be attributed to geographic, seasonal, or environmental factors, as noted by Kholif et al. (2023), which can influence the biochemical profile of pomegranate peel. The high TOM content (97.10% ± 2.84%) underscores PPE's suitability as a functional ingredient in edible coatings, providing a robust matrix for bioactive compound delivery (Ebadi et al. 2019; Khodanazary 2019; Khodanazary et al. 2019; Rahul et al. 2024). The elevated fiber content (10.55% ± 0.30% crude fiber, 29.52% ± 0.86% NDF, and 19.32% ± 0.56% ADF) enhances its potential as a dietary fiber source, which can improve gut health and texture in food applications (Raouf et al. 2022). The relatively low ash content (3.44% ± 0.10%) aligns with its minimal mineral residue, which is advantageous for maintaining sensory attributes in coated products. The observed variability in composition, as highlighted by Kholif et al. (2023), may stem from differences in cultivar, soil conditions, or extraction methods, necessitating standardized protocols for consistent PPE production (Mo et al. 2022). These findings suggest that PPE's composition supports its use as a sustainable, nutrient‐dense additive in food preservation systems. These compositions justify PPE's role as a nutrient‐dense additive, enhancing the nutritional profile of coated shrimp while providing functional benefits (Table 4).

**TABLE 3:** Approximate composition of pomegranate (*Punica granatum L.*) peel extract.

Chemical composition	Percentage (%)
Moisture	9.90 ± 0.29
Total organic matter (TOM)	97.10 ± 2.84
Protein	6.55 ± 0.19
Crude fiber	10.55 ± 0.30
Lipid	3.47 ± 0.10
Ash	3.44 ± 0.10
Nitrogen‐free extract (NFE)	76.50 ± 2.24
Neutral detergent fiber (NDF)	29.52 ± 0.86
Acid detergent fiber (ADF)	19.32 ± 0.56
Acid detergent lignin (ADL)	3.92 ± 0.11

**TABLE 4:** Optimum conditions of microencapsulation efficiency of PPE biological compounds samples.

Content	Temperature (°C)	Polyphenolic compound's microencapsulation percentage	Anthocyanin microencapsulation percentage
Freeze dry PPE sample	100	93.40 ± 0.74	100.00 ± 0.00
Freeze dry PPE sample	125	84.42 ± 0.89	91.92 ± 0.65
Freeze dry PPE sample	150	73.43 ± 0.23	85.07 ± 0.75

Table 5 presents the mineral profile of PPE, with significant differences (*p* < 0.05) observed in calcium (Ca: 343.88 ± 10.07 mg/100 g), phosphorus (P: 120.66 ± 3.53 mg/100 g), potassium (K: 150.83 ± 4.41 mg/100 g), sodium (Na: 68.37 ± 2.00 mg/100 g), and magnesium (Mg: 56.31 ± 1.64 mg/100 g). Trace elements included zinc (Z: 1.08 ± 0.03 mg/100 g), manganese (Mn: 0.86 ± 0.02 mg/100 g), copper (Cu: 0.65 ± 0.01 mg/100 g), iron (Fe: 6.14 ± 0.17 mg/100 g), and selenium (Se: 1.07 ± 0.03 mg/100 g). These results confirm PPE as a rich source of macro‐ and micro‐elements, contributing to its nutritional and functional value. El‐Beltagi et al. (2022) reported similar high levels of Ca, Mg, Fe, and Cu in PPE, corroborating these findings. The mineral content supports PPE's health benefits, including lowering LDL cholesterol, improving glucose resistance, and reducing cancer risk, as noted by Rowayshed et al. (2013). The significant mineral content of PPE, particularly Ca, K, and P, positions it as a valuable fortificant in food systems, enhancing nutritional quality and supporting metabolic functions (Spanova and Daum 2011). The high Ca (343.88 ± 10.07 mg/100 g) and K (150.83 ± 4.41 mg/100 g) levels may contribute to bone health and cardiovascular function, respectively, when incorporated into edible coatings (Siddiqui et al. 2024). The presence of trace elements like Fe and Se further enhances PPE's antioxidant capacity, as these minerals act as cofactors in enzymatic defense mechanisms against oxidative stress (Mo et al. 2022). The observed statistical significance (*p* < 0.05) in mineral content suggests robust analytical precision, though environmental factors, such as soil composition, may influence mineral accumulation, as noted by Kholif et al. (2023). These attributes make PPE an effective component in edible coatings for seafood, where mineral fortification can enhance both preservation and nutritional value. The high mineral content in PPE likely contributes to its stabilizing effect on shrimp pH and texture by influencing enzymatic activities during storage.

**TABLE 5:** Mineral compounds of pomegranate *(Punica granatum L.*) peel extract.

Mineral index	mg/100 g
Ca	343.88 ± 10.07
P	120.66 ± 3.53
K	150.83 ± 4.41
Na	68.37 ± 2.00
Mg	56.31 ± 1.64
Z	1.08 ± 0.03
Mn	0.86 ± 0.02
Cu	0.65 ± 0.01
Fe	6.14 ± 0.17
Se	1.07 ± 0.03

The fatty acid profile of PPE (Table 6) revealed docosahexaenoic acid (DHA: 17.31% ± 0.50%), linoleic acid (13.96% ± 0.40%), eicosapentaenoic acid (EPA: 12.29% ± 0.36%), palmitic acid (11.50% ± 0.36%), and elaidic acid (11.01% ± 0.32%) as the primary fatty acids. These results highlight a significant presence of omega‐3 polyunsaturated fatty acids (PUFAs), such as EPA and DHA, alongside saturated and monounsaturated fatty acids. Akuru et al. (2022) reported a similar fatty acid composition, with stearic acid as the dominant saturated fatty acid, while Andrés et al. (2017) noted improved meat quality with pomegranate by‐products, consistent with this study's findings. The omega‐3 fatty acids EPA and DHA are known to support gut, heart, and brain health (Chen and Liu 2020; Elbaz 2023). The predominance of DHA (17.31% ± 0.50%) and EPA (12.29% ± 0.36%) in PPE underscores its potential to enhance the nutritional profile of coated shrimp, given the well‐documented benefits of omega‐3 PUFAs in reducing inflammation and supporting cardiovascular health (Kharchoufi et al. 2018). The presence of linoleic acid (13.96% ± 0.40%), an essential omega‐6 fatty acid, further contributes to PPE's functional properties, potentially improving lipid stability in seafood products. The balance of saturated (e.g., palmitic acid: 11.50% ± 0.36%) and unsaturated fatty acids suggests PPE's role in maintaining oxidative stability during storage, as PUFAs are prone to oxidation but can be protected by PPE's phenolic compounds (Ebadi et al. 2019; Khodanazary 2019; Khodanazary et al. 2019; Rahul et al. 2024). Variations in fatty acid profiles, as noted by Akuru et al. (2022), may arise from extraction techniques or peel maturity, warranting further optimisation of processing methods to maximize PUFA retention. These findings support PPE's application in edible coatings to enhance both the nutritional and shelf‐life quality of shrimp. This profile explains the observed preservation of PUFAs in treated shrimp, as PPE's antioxidants protect against oxidation, unlike controls where degradation was faster due to unrestricted lipid peroxidation.

**TABLE 6:** Fatty acid profile of pomegranate (*Punica granatum L.*) peel extract.

Fatty acid profile index	Percentage (%)
Capric acid (C10)	1.02 ± 0.03
Lauric acid (C12)	0.83 ± 0.02
Myristic acid (C14)	1.09 ± 0.03
Pentadecanoic acid (C15)	2.03 ± 0.06
Palmitic acid (C16)	11.50 ± 0.36
Palmitoleic acid (C16:1N7)	3.13 ± 0.09
Palmitoleic acid (C16:1N9)	1.65 ± 0.04
Margaric acid (C17)	0.75 ± 0.02
Stearic acid (C18)	3.74 ± 0.10
Elaidic acid (C18:1N9)	11.01 ± 0.32
Linoleic acid (C18:2N6 Trans)	1.84 ± 0.05
Linoleic acid (C18:2N6 Cis)	13.96 ± 0.40
α‐linoleic acid (C18:3N3)	6.06 ± 0.17
Eicosenoic acid (C20:1N9)	1.44 ± 0.04
Dihomo‐γ‐linolenic acid (C20:3N6)	1.75 ± 0.05
Arachidonic acid (C20:4N6)	3.26 ± 0.09
Eicosapentaenoic acid (EPA) (C20:5N3)	12.29 ± 0.36
Heneicosanoic acid (C21)	1.17 ± 0.03
Docosahexaenoic acid (DHA) (C22:6N3)	17.31 ± 0.50
Nervonic acid (C24:1N9)	3.61 ± 0.10

Table 7 outlines the phenolic composition of PPE, with catechins (896.91 ± 26.26 mg/100 g), phenols (255.75 ± 7.48 mg/100 g), gallic acid (128.80 ± 3.77 mg/100 g), and ellagic acid (52.31 ± 1.53 mg/100 g) as the major compounds. Other phenolics included resorcinol (14.16 ± 0.41 mg/100 g), protocatechol (4.64 ± 0.13 mg/100 g), p‐hydroxybenzoic acid (10.38 ± 0.30 mg/100 g), vanillin (4.19 ± 0.12 mg/100 g), caffeic acid (55.53 ± 1.62 mg/100 g), ferulic acid (6.14 ± 0.17 mg/100 g), p‐coumaric acid (14.29 ± 0.41 mg/100 g), and others (5.16 ± 0.15 mg/100 g). These compounds contribute to PPE's antioxidant and anti‐inflammatory properties. Das et al. (2021) and Hussein et al. (2022) confirmed high levels of ellagic acid, punicalagin, and catechins in PPE, supporting its efficacy in food preservation. The antimicrobial and anticancer benefits of these phenolics, including flavonoids and tannins, are well‐documented (Belgacem et al. 2021; Gullón et al. 2020). The high catechin content (896.91 ± 26.26 mg/100 g) and significant levels of gallic and ellagic acids highlight PPE's potent antioxidant capacity, which is critical for inhibiting lipid oxidation in shrimp during refrigerated storage (Teniente et al. 2023). These phenolics, particularly catechins and ellagic acid, disrupt microbial cell membranes and scavenge free radicals, enhancing food safety and shelf life (Gullón et al. 2020). The presence of caffeic and ferulic acids further contributes to PPE's antimicrobial properties, as these compounds inhibit bacterial enzyme activity (Siddiqui et al. 2024). The variability in phenolic content, as noted by Hussein et al. (2022), may be influenced by extraction solvents or peel processing, suggesting a need for optimized extraction protocols to maximize bioactive yield. These findings validate PPE's role as a natural preservative in edible coatings, offering both functional and health benefits. The high phenolic levels correlate with reduced TBARS and microbial counts in T1 and T2, as these compounds chelate metals and disrupt bacterial membranes, providing a mechanistic basis for the superior performance of encapsulated PPE.

**TABLE 7:** Phenolic compounds of pomegranate (*Punica granatum L.*) peel extract.

Phenolic compounds	mg/100 g
Ellagic acid	52.31 ± 1.53
Catechins	896.91 ± 26.26
Gallic acid	128.80 ± 3.77
Resocenol	14.16 ± 0.41
Protocatechol	4.64 ± 0.13
р‐hydroxy benzoic acid	10.38 ± 0.30
Phenol	255.75 ± 7.48
Vanillin	4.19 ± 0.12
Caffeic acid	55.53 ± 1.62
Ferulic acid	6.14 ± 0.17
р‐coumaric acid	14.29 ± 0.41
Others	5.16 ± 0.15

The vitamin profile of PPE (Table 8) revealed vitamin C (13.33 ± 0.39 mg/100 g) and vitamin E (4.15 ± 0.12 mg/100 g) as the most abundant, followed by vitamin A (0.18 ± 0.01 mg/100 g), vitamin B1 (0.14 ± 0.00 mg/100 g), and vitamin B2 (0.09 ± 0.00 mg/100 g). These vitamins contribute to PPE's antioxidant capacity and nutritional value. Omer et al. (2019) reported similar vitamin profiles, reinforcing PPE's role as a valuable source of antioxidants. The presence of vitamins C and E supports PPE's ability to mitigate oxidative stress in food systems (Magangana et al. 2020). The elevated levels of vitamin C (13.33 ± 0.39 mg/100 g) and vitamin E (4.15 ± 0.12 mg/100 g) enhance PPE's antioxidant capacity, complementing its phenolic compounds in preventing lipid peroxidation in shrimp (Mo et al. 2022). Vitamin C, a potent free radical scavenger, and vitamin E, a lipid‐soluble antioxidant, work synergistically to protect cellular membranes, thereby extending shelf life (Kharchoufi et al. 2018). The lower levels of vitamins A, B1, and B2 still contribute to PPE's nutritional profile, supporting its use in functional foods. The consistency with Omer et al. (2019) suggests reliable vitamin retention in PPE, though processing conditions, such as heat or pH, may affect vitamin stability, warranting further investigation (Siddiqui et al. 2024). These attributes make PPE an effective component in edible coatings for enhancing both preservation and nutritional quality. Vitamins C and E in PPE likely synergise with polyphenols to enhance antioxidant capacity, explaining the delayed onset of rancidity in coated samples compared to previous studies using vitamin‐deficient preservatives.

**TABLE 8:** Vitamin composition of pomegranate (*Punica granatum L.*) peel extraction.

Vitamin compounds	mg/100 g
Vitamin B1 (Thiamin)	0.14 ± 0.00
Vitamin B2 (Riboflavin)	0.09 ± 0.00
Vitamin C (Ascorbic acid)	13.33 ± 0.39
Vitamin E (α‐tocopherol)	4.15 ± 0.12
Vitamin A (Retinol)	0.18 ± 0.01

Table 9 demonstrates the antibacterial and antioxidant properties of PPE. The minimum inhibitory concentration (MIC) for 0.3% PPE was significantly higher (*p* < 0.05) against *Escherichia coli* (20.77 ± 0.33 mm) and *Staphylococcus aureus* (14.60 ± 0.02 mm) compared to 0.1% PPE ( *E. coli* : 15.29 ± 0.22 mm; *S. aureus* : 12.91 ± 0.23 mm) and potassium sorbate ( *E. coli* : 13.02 ± 0.19 mm; *S. aureus* : 12.73 ± 0.23 mm). The control (70% ethanol) showed no inhibition. Antioxidant activity, measured via DPPH (82.02% ± 1.65%) and FRAP (1531.99 ± 51 mmol Fe/L), confirmed PPE's high antioxidant capacity. Abdel‐Aziz et al. (2021) reported strong antibacterial activity of PPE against *E. coli* , consistent with these findings. Ismail et al. (2019) noted that ethanolic PPE extracts were particularly effective against *Bacillus cereus* and *S. aureus* in shrimp, supporting its preservative potential. The superior MIC values of 0.3% PPE (*p* < 0.05) against *E. coli* and *S. aureus* highlight its potent antimicrobial activity, likely due to its high phenolic content, particularly catechins and ellagic acid, which disrupt bacterial cell walls (Ghasemi et al. 2023). The higher efficacy of 0.3% PPE compared to potassium sorbate suggests its potential as a natural alternative to synthetic preservatives, aligning with consumer demand for clean‐label products (Siddiqui et al. 2024). The DPPH (82.02% ± 1.65%) and FRAP (1531.99 ± 51 mmol Fe/L) results underscore PPE's robust antioxidant capacity, attributed to its synergistic phenolic and vitamin content (Mo et al. 2022). These properties make PPE an effective component in edible coatings, reducing microbial growth and oxidative degradation in shrimp, as supported by Ismail et al. (2019). Further studies are needed to optimize PPE concentration and delivery systems to maximize antimicrobial efficacy without compromising sensory attributes. These properties justify the low TBC in T1 and T2, as encapsulation ensures sustained release, overcoming limitations in free‐form PPE where rapid degradation reduces efficacy (Nair et al. 2020).

**TABLE 9:** Antibacterial and antioxidant properties of pomegranate (*Punica granatum L.*) extract.

Abacterial factors	Minimum inhibitory concentration (MIC) (mm)
Ethanol 70% (control samples)	
*E. coli*	0
*Staphylococcus aureus*	0
Potassium sorbate 0.1%	
*E. coli*	13.02 ± 19
*Staphylococcus aureus*	12.73 ± 0.23
Pomegranate peel extraction 0.1% concentration	
*E. coli*	15.29 ± 0.22
*Staphylococcus aureus*	12.91 ± 0.23
Pomegranate peel extraction 0.3% concentration	
*E. coli*	20.77 ± 0.33
*Staphylococcus aureus*	14.60 ± 0.02
Antioxidant factors	
DPPH (%)	82.02 ± 1.65
FRAP (mmol Fe/L)	1531.99 ± 51

Table 10 details the physicochemical properties of shrimp treatments over 15 days. Lipid content showed no significant differences (*p* > 0.05), ranging from 12.34% ± 0.05% (T5, day 3) to 12.68% ± 0.25% (T3, day 12). The pH varied significantly (*p* < 0.05), with the lowest value in T1 (7.34 ± 0.02, day 3) and the highest in T5 (8.30 ± 0.03, day 9). Thiobarbituric acid reactive substances (TBARS) values, indicative of lipid oxidation, ranged from 0.18 ± 0.01 mg MDA/kg (T2, day 3) to 1.02 ± 0.03 mg MDA/kg (T5, day 9), with significant differences (*p* < 0.05). Basiri et al. (2015) reported similar pH trends, attributing increases to microbial activity and nitrogenous compound accumulation. Salehi et al. (2022) noted that PPE reduced TBARS levels, supporting its antioxidant efficacy. The stable lipid content across treatments (*p* > 0.05) suggests that the alginate‐gum arabic coating with PPE effectively maintained lipid integrity during storage, likely due to the antioxidant properties of PPE's phenolics and vitamins (Salehi et al. 2022). The significant pH increase in T5 (8.30 ± 0.03, day 9) indicates higher microbial activity or protein degradation, consistent with Basiri et al. (2015), but PPE‐treated samples (T1, T2) exhibited lower pH values, suggesting reduced microbial proliferation (Ebadi et al. 2019; Khodanazary 2019; Khodanazary et al. 2019; Rahul et al. 2024). The low TBARS values in T2 (0.18 ± 0.01 mg MDA/kg, day 3) reflect PPE's ability to inhibit lipid oxidation, attributed to its high catechin and vitamin C content (Siddiqui et al. 2024). These findings highlight the efficacy of PPE‐enriched coatings in maintaining physicochemical stability, though extended storage beyond 9 days may compromise quality due to rising pH and TBARS values. The lower TBARS in encapsulated treatments can be attributed to controlled polyphenol release, preventing hydroperoxide formation more effectively than free‐form PPE, as evidenced by slower pH rise linked to reduced amine production from bacterial proteolysis (Mehraie et al. 2023).

**TABLE 10:** Physiochemical properties of shrimp treatments during 15 days.

Days	Treatments	Lipid (%)	pH	Separated lipid quantity of samples (g)	TBARS (mg malonaldehyde/kg meat)
The 3rd day	T1	12.43 ± 0.10^bcA^	7.34 ± 0.02^dE^	0.21 ± 0.01^aA^	0.21 ± 0.01e^D^
	T2	12.58 ± 0.05^abA^	7.54 ± 0.10^cD^	0.19 ± 0.01^aA^	0.18 ± 0.01f^D^
	T3	12.64 ± 0.25^aA^	7.89 ± 0.05^aE^	0.20 ± 0.01^aA^	0.28 ± 0.01^cD^
	T4	12.52 ± 0.12^abcA^	7.89 ± 0.01^aC^	0.17 ± 0.01^bA^	0.24 ± 0.01^dD^
	T5	12.34 ± 0.05^cA^	8.10 ± 0.05^aB^	0.14 ± 0.01^cA^	0.34 ± 0.02^bC^
	T6	12.46 ± 0.08^abcA^	7.71 ± 0.2^bB^	0.22 ± 0.00^aA^	0.38 ± 0.03^aB^
The 6th day	T1	12.46 ± 0.10^bcA^	7.46 ± 0.02^eD^	0.29 ± 0.01^aB^	0.29 ± 0.01^eC^
	T2	12.61 ± 0.05^abA^	7.66 ± 0.10^dC^	0.27 ± 0.01^aB^	0.26 ± 0.01f^C^
	T3	12.67 ± 0.25^aA^	8.11 ± 0.05^bD^	0.28 ± 0.01^aB^	0.38 ± 0.25^cC^
	T4	12.55 ± 0.12^abcA^	8.11 ± 0.01^bB^	0.25 ± 0.01^bB^	0.35 ± 0.01^dC^
	T5	12.37 ± 0.05^cA^	8.25 ± 0.05^aA^	0.22 ± 0.01^cB^	0.59 ± 0.03^bB^
	T6	12.49 ± 0.08^abcA^	8.02 ± 0.02^cA^	0.30 ± 0.00^aB^	0.92 ± 0.01^aA^
The 9th day	T1	12.44 ± 0.10^cA^	7.51 ± 0.02^dC^	0.35 ± 0.01^aC^	0.42 ± 0.01^dB^
	T2	12.59 ± 0.05^abA^	7.71 ± 0.10^cB^	0.33 ± 0.01^aC^	0.39 ± 0.01^eBC^
	T3	12.65 ± 0.25^aA^	8.16 ± 0.05^bC^	0.34 ± 0.01^aC^	0.51 ± 0.03^bB^
	T4	12.53 ± 0.12^bA^	8.16 ± 0.01^bB^	0.31 ± 0.01^bC^	0.48 ± 0.01^cB^
	T5	12.35 ± 0.05^cA^	8.30 ± 0.03^aA^	0.28 ± 0.01^cC^	1.02 ± 0.03^aA^
The 12th day	T1	12.47 ± 0.10^dA^	7.64 ± 0.02^cB^	0.42 ± 0.01^aD^	0.50 ± 0.01^cB^
	T2	12.62 ± 0.05^bA^	7.84 ± 0.10^bA^	0.40 ± 0.01^aD^	0.47 ± 0.01^dB^
	T3	12.68 ± 0.25^aA^	8.23 ± 0.05^aB^	0.41 ± 0.01^aD^	0.59 ± 0.03^aB^
	T4	12.56 ± 0.12^cA^	8.23 ± 0.01^aA^	0.38 ± 0.01^bD^	0.56 ± 0.01^bB^
The 15th day	T1	12.45 ± 0.10^dA^	7.69 ± 0.02^cA^	0.50 ± 0.01^aE^	0.61 ± 0.01^cA^
	T2	12.60 ± 0.05^bA^	7.89 ± 0.10^bA^	0.48 ± 0.01^aE^	0.58 ± 0.01^dA^
	T3	12.66 ± 0.25^aA^	8.28 ± 0.05^aA^	0.49 ± 0.01^aE^	0.75 ± 0.03^aA^
	T4	12.54 ± 0.12^cA^	8.28 ± 0.01^aA^	0.46 ± 0.01^bE^	0.72 ± 0.01^bA^

*Note:* The letters a, b, … in each column indicate significant differences among treatments on the same day. The letters A, B, … indicate significant differences for each treatment over 15 days.

Table 11 shows texture parameters over 15 days. Firmness and resilience were highest on day 3 (e.g., T5: 4.75 ± 0.06 g firmness; T1: 1.18 ± 0.07 resilience) and lowest on day 15 (e.g., T4: 2.98 ± 0.18 g firmness; T1: 1.03 ± 0.06 resilience). Cohesiveness and adhesiveness showed no significant differences (*p* > 0.05), though values were slightly higher on day 15. Hung et al. (2023) reported similar texture retention in treated shrimp, attributing it to reduced microbial activity and protein degradation. The decline in firmness and resilience over 15 days reflects natural protein denaturation and water loss in shrimp, but PPE‐treated samples (T1, T2) maintained higher values compared to controls, likely due to PPE's antimicrobial properties reducing proteolytic bacterial activity (Hung et al. 2023). The lack of significant differences in cohesiveness and adhesiveness (*p* > 0.05) suggests that the alginate‐gum arabic coating provided structural stability, preventing excessive texture degradation (Kharchoufi et al. 2018). The incorporation of PPE's phenolics, particularly tannins, may have cross‐linked with shrimp proteins, enhancing texture retention (Ebadi et al. 2019; Khodanazary 2019; Khodanazary et al. 2019; Rahul et al. 2024). These results underscore the coating's efficacy in preserving shrimp texture, though further studies could explore optimal coating thickness to minimize adhesiveness variations. The maintenance of firmness in T1 and T2 is likely due to PPE's inhibition of proteolytic enzymes and moisture loss prevention by the coating, contrasting with controls where autolysis and bacterial degradation accelerated softening (Dehghani et al. 2018).

**TABLE 11:** Texture of shrimp treatments during 15 days.

Days	Treatments	Firmness (g)	Cohesiveness (g)	Resilience	Adhesiveness (g/s)
The 3rd day	T1	3.75 ± 0.24^cA^	0.51 ± 0.00^cC^	1.18 ± 0.07^dA^	1.80 ± 0.01^bD^
	T2	3.67 ± 0.06^cA^	0.52 ± 0.00^cC^	1.29 ± 0.04^dA^	1.86 ± 0.00^bD^
	T3	3.47 ± 0.24^cA^	0.58 ± 0.01^bB^	1.62 ± 0.07^cA^	1.45 ± 0.23^cC^
	T4	3.60 ± 0.02^cA^	0.62 ± 0.00^aC^	1.81 ± 0.07^bA^	1.66 ± 0.04^cC^
	T5	4.75 ± 0.06^aA^	0.62 ± 0.03^aA^	1.42 ± 0.19^cA^	2.02 ± 0.20^aB^
	T6	3.96 ± 0.12^b^	0.53 ± 0.00^c^	1.91 ± 0.09^a^	1.20 ± 0.05^d^
The 6th day	T1	3.60 ± 0.23^bB^	0.54 ± 0.00^cBC^	1.15 ± 0.06^dAB^	2.02 ± 0.01^bC^
	T2	3.52 ± 0.06^bB^	0.55 ± 0.01^cB^	1.25 ± 0.04^cAB^	2.08 ± 0.00^bC^
	T3	3.33 ± 0.23^bB^	0.61 ± 0.01^bB^	1.57 ± 0.07^bB^	1.63 ± 0.26^dBC^
	T4	3.46 ± 0.02^bB^	0.66 ± 0.00^aB^	1.75 ± 0.06^aB^	1.86 ± 0.04^cC^
	T5	4.38 ± 0.06^aB^	0.65 ± 0.04^aA^	1.38 ± 0.18^cA^	2.26 ± 0.22^aA^
The 9th day	T1	3.45 ± 0.22^aC^	0.56 ± 0.01^cB^	1.11 ± 0.06^cB^	2.26 ± 0.01^aB^
	T2	3.38 ± 0.06^aC^	0.57 ± 0.00^cB^	1.21 ± 0.04^cB^	2.33 ± 0.00^aB^
	T3	3.20 ± 0.22^aC^	0.63 ± 0.01^bB^	1.53 ± 0.06^bB^	1.82 ± 0.29^cB^
	T4	3.32 ± 0.02^aC^	0.68 ± 0.00^aB^	1.70 ± 0.06^aB^	2.08 ± 0.05^bB^
The 12th day	T1	3.31 ± 0.21^aD^	0.59 ± 0.00^cB^	1.08 ± 0.06^cB^	2.53 ± 0.01^aAB^
	T2	3.25 ± 0.05^aC^	0.60 ± 0.01^cB^	1.18 ± 0.04^cB^	2.61 ± 0.01^aA^
	T3	3.07 ± 0.21^aD^	0.67 ± 0.01^bA^	1.48 ± 0.06^bB^	2.04 ± 0.33^cB^
	T4	3.19 ± 0.02^aD^	0.72 ± 0.00^aA^	1.65 ± 0.06^aB^	2.29 ± 0.01^bA^
The 15th day	T1	3.25 ± 0.20^aD^	0.65 ± 0.00^bA^	1.03 ± 0.06^cC^	2.65 ± 0.01^bA^
	T2	3.32 ± 0.11^aC^	0.65 ± 0.00^bA^	1.08 ± 0.01^bC^	2.70 ± 0.05^aA^
	T3	3.18 ± 0.05^aC^	0.66 ± 0.01^bA^	1.12 ± 0.03^bC^	2.74 ± 0.01^aA^
	T4	2.98 ± 0.18^aE^	0.71 ± 0.04^aA^	1.25 ± 0.17^aC^	2.59 ± 0.17^bA^

*Note:* The letters a, b, … in each column indicate significant differences among treatments on the same day. The letters A, B, … indicate significant differences for each treatment over 15 days.

Table 12 presents sensory evaluation results, with T1 and T2 scoring highest for taste, texture, color, odor, and overall acceptance across 15 days. Scores decreased over time, with T1 and T2 maintaining significantly higher values (*p* < 0.05) than T3 and T4 by day 15 (e.g., T1: 3.23 ± 0.42 overall acceptance; T4: 2.53 ± 0.66). Abbas and Abdul‐Rahman (2020) and Elsayed (2023) reported improved sensory attributes with PPE, attributed to its antimicrobial and antioxidant properties. The superior sensory scores of T1 and T2 (*p* < 0.05) reflect the effectiveness of PPE‐enriched coatings in preserving shrimp's sensory attributes, likely due to reduced lipid oxidation and microbial growth (Elsayed 2023). The high catechin and ellagic acid content in PPE inhibited off‐odors and color degradation, maintaining consumer acceptability (Siddiqui et al. 2024). The decline in scores by day 15, particularly in T3 and T4, aligns with increased pH and TBARS values, indicating oxidative and microbial deterioration (Ebadi et al. 2019; Khodanazary 2019; Khodanazary et al. 2019; Rahul et al. 2024). These findings suggest that PPE at 0.3% concentration (T1, T2) optimizes sensory quality, though sensory thresholds for PPE concentration should be further investigated to avoid potential bitterness from high phenolic levels. Higher scores in encapsulated treatments reflect masked off‐flavors from oxidation and spoilage, with the coating providing a neutral barrier that preserves natural shrimp attributes longer than uncoated samples (Yuan et al. 2016).

**TABLE 12:** Sensory evaluation of shrimp treatments during 15 days.

Days	Treatments	Taste	Texture	Color	Odors	Total acceptance
The 3rd day	T1	5.00 ± 0.00^aA^	5.00 ± 0.00^aA^	5.00 ± 0.00^aA^	5.00 ± 0.00^aA^	5.00 ± 0.00^aA^
	T2	5.00 ± 0.00^aA^	5.00 ± 0.00^a^	5.00 ± 0.00^aA^	5.00 ± 0.00^aA^	4.76 ± 0.43^bA^
	T3	4.84 ± 0.37^aA^	5.00 ± 0.00^a^	4.53 ± 0.51^bA^	5.00 ± 0.00^aA^	4.61 ± 0.50^bA^
	T4	4.92 ± 0.27^aA^	5.00 ± 0.00^a^	4.61 ± 0.51^bA^	4.76 ± 0.43^abA^	4.76 ± 0.43^bA^
	T5	3.92 ± 0.27^bA^	5.00 ± 0.00^aA^	4.15 ± 0.37^cA^	3.76 ± 0.59^cA^	4.31 ± 0.48^cA^
	T6	4.84 ± 0.37^a^	5.00 ± 0.00^a^	5.00 ± 0.00^a^	4.00 ± 0.40^b^	4.92 ± 0.27^a^
The 6th day	T1	4.92 ± 0.27^aA^	4.92 ± 0.27^aA^	5.00 ± 0.00^aA^	4.92 ± 0.27^aA^	4.92 ± 0.27^aA^
	T2	4.92 ± 0.27^aA^	4.92 ± 0.27^a^	4.92 ± 0.27^aA^	5.00 ± 0.00^aA^	4.69 ± 0.48^bA^
	T3	4.76 ± 0.43^aA^	4.92 ± 0.27^a^	4.53 ± 0.51^bA^	5.00 ± 0.00^aA^	4.61 ± 0.50^bA^
	T4	4.76 ± 0.43^aA^	4.92 ± 0.27^a^	4.53 ± 0.51^bA^	4.69 ± 0.48^bA^	4.69 ± 0.48^bA^
	T5	3.69 ± 0.48^bA^	4.76 ± 0.43^aA^	3.84 ± 0.55^cB^	3.61 ± 0.50^cA^	4.07 ± 0.27^cA^
The 9th day	T1	4.69 ± 0.47^aA^	4.76 ± 0.42^aAB^	4.76 ± 0.42^aA^	4.61 ± 0.49^aA^	4.69 ± 0.47^aA^
	T2	4.61 ± 0.50^aA^	4.69 ± 0.48^a^	4.69 ± 0.48^aAB^	4.76 ± 0.43^aA^	4.46 ± 0.51^abcAB^
	T3	4.23 ± 0.59^bA^	4.46 ± 0.51^b^	3.76 ± 0.92^cB^	4.46 ± 0.51^abAB^	4.15 ± 0.55^abcB^
	T4	4.38 ± 0.50^bAB^	4.38 ± 0.51^b^	4.23 ± 0.59^bA^	3.92 ± 0.95^abcB^	4.30 ± 0.48^abcB^
The 12th day	T1	4.15 ± 0.67^aAB^	4.30 ± 0.73^abAB^	4.31 ± 0.74^aAB^	4.07 ± 0.84^bAB^	4.23 ± 0.42^aB^
	T2	4.23 ± 0.72^aA^	4.53 ± 0.51^a^	4.38 ± 0.50^aAB^	4.46 ± 0.51^aAB^	4.15 ± 0.37^aB^
	T3	3.92 ± 0.86^abB^	4.07 ± 0.64^ab^	3.69 ± 0.94^bB^	4.23 ± 0.83^bAB^	3.76 ± 0.59^bC^
	T4	3.76 ± 0.8^abC^	4.15 ± 0.80^ab^	3.69 ± 0.75^bB^	3.69 ± 0.94^cB^	3.53 ± 0.66^bC^
The 15th day	T1	3.15 ± 0.67^aB^	3.31 ± 0.73^aC^	3.31 ± 0.73^aC^	3.07 ± 0.84^aC^	3.23 ± 0.42^aC^
	T2	3.23 ± 0.72^aB^	3.53 ± 0.51^a^	3.38 ± 0.50^aC^	3.46 ± 0.51^aC^	3.15 ± 0.37^aC^
	T3	2.92 ± 0.86^aC^	3.07 ± 0.64^a^	2.69 ± 0.94^aC^	3.23 ± 0.83^aC^	2.76 ± 0.59^abD^
	T4	2.76 ± 0.83^aD^	3.15 ± 0.80^a^	2.69 ± 0.75^aC^	2.69 ± 0.94^aC^	2.53 ± 0.66^abD^

*Note:* The letters a, b, … in each column indicate significant differences among treatments on the same day. The letters A, B, … indicate significant differences for each treatment over 15 days.

Table 13 shows bacterial growth, with the lowest total bacterial count (TBC) in T1 (5.50 ± 0.57 log CFU/g) and T2 (5.00 ± 0.00 log CFU/g) on day 3, maintaining significantly lower counts (*p* < 0.05) than T3 and T4 by day 15 (e.g., T3: 14.50 ± 1.73 log CFU/g; T4: 14.50 ± 0.57 log CFU/g). Specific pathogens, including *E. coli* and *S. aureus* , followed similar trends. Elsayed (2023) reported that 3% PPE significantly reduced microbial growth, consistent with these findings. PPE's tannins disrupt microbial cell walls, enhancing preservation (Miguel et al. 2010). The significantly lower TBC in T1 and T2 (*p* < 0.05) demonstrates the potent antimicrobial activity of PPE, particularly at 0.3% concentration, which outperformed controls and lower concentrations (T3, T4). The phenolic compounds, especially tannins and catechins, likely disrupted bacterial cell membranes, as noted by Miguel et al. (2010), while the alginate‐gum arabic coating provided a physical barrier to microbial penetration (Ebadi et al. 2019; Khodanazary 2019; Khodanazary et al. 2019; Rahul et al. 2024). The increased TBC in T3 and T4 by day 15 reflects reduced PPE efficacy at lower concentrations, highlighting the dose‐dependent antimicrobial effect (Siddiqui et al. 2024). These results support PPE's role in extending shrimp shelf life, though further studies could explore synergistic antimicrobial agents to enhance efficacy at lower PPE concentrations. The superior microbial control in T1 and T2 stems from encapsulation's protection against degradation, allowing prolonged antimicrobial action against psychrotrophs like Pseudomonas, unlike free‐form where efficacy wanes after day 9 (Ismail et al. 2019).

**TABLE 13:** Bacterial growth of shrimp treatments during 15 days.

Days	Treatments	Total bacterial count (TBC)	Cryophilic bacteria's	*Staphylococcus aureus*	*E. coli*
The 3rd day	T1	5.50 ± 0.57^cD^	4.00 ± 0.00^cE^	2.00 ± 0.00^aE^	1.5 ± 0.57^bD^
	T2	5.00 ± 0.00^cE^	3.50 ± 0.57^cE^	2.00 ± 0.00^aD^	1.00 ± 0.00^cE^
	T3	6.50 ± 0.58^bE^	6.50 ± 0.58^aE^	2.00 ± 0.00^aE^	2.50 ± 0.58^abE^
	T4	5.50 ± 0.57^cE^	5.00 ± 0.00^bE^	2.00 ± 0.00^a^	1.50 ± 0.57^bE^
	T5	7.00 ± 0.00^bB^	5.50 ± 0.57^abB^	2.50 ± 0.57^abC^	2.50 ± 0.58^abC^
	T6	7.5 ± 0.58^aB^	6.50 ± 0.58^aB^	2.50 ± 0.58^abB^	3.50 ± 0.57^aB^
The 6th day	T1	6.50 ± 0.57^cC^	5.00 ± 0.00^cD^	3.00 ± 0.00^bD^	2.50 ± 0.58^bcC^
	T2	6.00 ± 0.00^cD^	4.50 ± 0.57^cD^	3.00 ± 0.00^bC^	1.50 ± 0.57^dD^
	T3	7.50 ± 0.58^cD^	8.50 ± 0.57^bD^	3.50 ± 0.57^abD^	3.00 ± 0.00^bCDE^
	T4	6.50 ± 0.57^cD^	7.00 ± 0.00^bD^	4.00 ± 0.00^b^	2.00 ± 1.15^cCDE^
	T5	9.00 ± 0.00^bA^	8.50 ± 0.58^bA^	4.50 ± 0.58^bB^	3.50 ± 0.58^bB^
	T6	17.00 ± 1.15^aA^	12.50 ± 0.57^aA^	9.00 ± 1.15^aA^	5.50 ± 0.57^aA^
The 9th day	T1	7.00 ± 0.00^bC^	6.00 ± 0.00^bC^	4.00 ± 0.00^bBC^	3.00 ± 0.00^bBC^
	T2	7.00 ± 0.00^bC^	5.50 ± 0.58^bC^	4.00 ± 0.00^bB^	2.50 ± 0.58^bcC^
	T3	8.50 ± 0.58^bC^	9.50 ± 0.57^aC^	4.50 ± 0.57^abBC^	4.50 ± 0.57^aC^
	T4	7.50 ± 0.57^bC^	8.00 ± 0.00^aC^	5.00 ± 0.00^a^	2.50 ± 0.58^bcC^
	T5	10.00 ± 0.00^aA^	9.50 ± 0.58^aA^	5.50 ± 0.58^aA^	5.00 ± 0.00^aA^
The 12th day	T1	8.00 ± 0.00^aB^	7.00 ± 0.00^bB^	4.50 ± 0.58^bB^	3.50 ± 0.57^bB^
	T2	8.00 ± 0.00^aB^	6.50 ± 0.57^bB^	4.00 ± 0.00^bB^	3.00 ± 0.00^bABC^
	T3	9.50 ± 0.58^aB^	10.50 ± 0.58^aB^	5.00 ± 0.00^aB^	5.50 ± 0.58^aB^
	T4	8.50 ± 0.57^aB^	9.00 ± 0.00^aB^	5.00 ± 0.00^a^	3.50 ± 0.57^bB^
The 15th day	T1	10.50 ± 0.58^bA^	8.00 ± 0.00^bA^	5.50 ± 0.57^bA^	4.50 ± 0.58^bA^
	T2	10.00 ± 0.00^bA^	7.50 ± 0.57^bA^	5.00 ± 0.00^bA^	3.50 ± 0.57^bcA^
	T3	14.50 ± 1.73^aA^	11.50 ± 0.58^aA^	6.00 ± 0.00^aA^	6.00 ± 1.15^aA^
	T4	14.50 ± 0.57^aA^	10.50 ± 0.57^aA^	6.00 ± 0.00^a^	4.50 ± 0.58^bA^

*Note:* The letters a, b, … in each column indicate significant differences among treatments on the same day. The letters A, B, … indicate significant differences for each treatment over 15 days.

**FIGURE 3:**
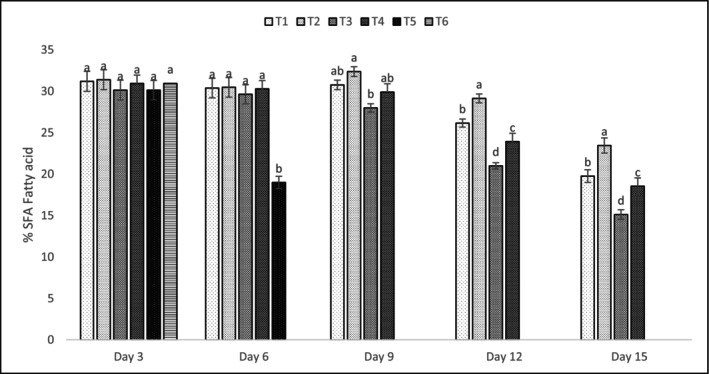
Saturated fatty acid (SFA) graph of shrimp treatments over 15 days. The letters a, b, … in each column indicate significant differences among treatments on the same day. The letters A, B, … indicate significant differences for each treatment over 15 days.

**FIGURE 4:**
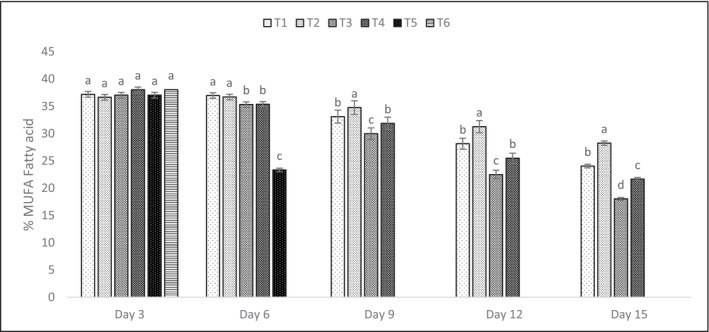
Monounsaturated fatty acid (MUFA) graph of shrimp treatments over 15 days. The letters a, b, … in each column indicate significant differences among treatments on the same day. The letters A, B, … indicate significant differences for each treatment over 15 days.

**FIGURE 5:**
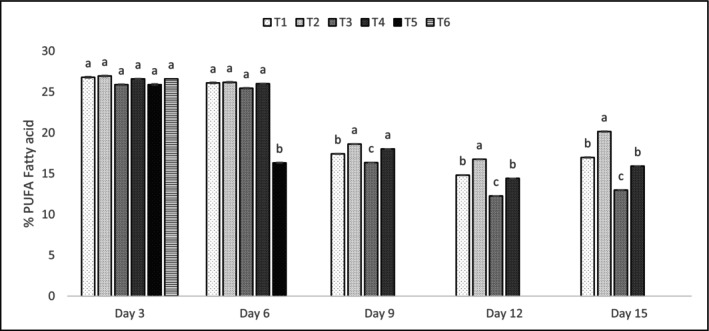
Polyunsaturated fatty acid (PUFA) graph of shrimp treatments over 15 days. The letters a, b, … in each column indicate significant differences among treatments on the same day. The letters A, B, … indicate significant differences for each treatment over 15 days.

Tables 14, 15, 16 and Figures 3, 4, 5 detail the fatty acid profiles of shrimp treatments. Saturated fatty acids (SFAs) were highest on day 3 (e.g., T4: 20.91% ± 0.80% palmitic acid), with significant declines by day 15 (*p* < 0.05). Monounsaturated fatty acids (MUFAs) followed a similar trend, with elaidic acid (26.91% ± 0.77% in T4, day 3) being dominant. Polyunsaturated fatty acids (PUFAs), including DHA (11.38% ± 0.89% in T2, day 3) and EPA (5.10% ± 0.78% in T4, day 3), were best preserved in T1 and T2, with significant differences (*p* < 0.05) compared to T3 and T4 by day 15. Naczk and Shahidi (2004) noted that PPE preserved PUFA levels, particularly EPA and DHA, during storage, consistent with these findings. The preservation of PUFAs, particularly DHA and EPA, in T1 and T2 (*p* < 0.05) highlights the antioxidant efficacy of PPE‐enriched coatings, which protected against lipid oxidation, as supported by Naczk and Shahidi (2004). The decline in SFAs and MUFAs by day 15 reflects natural lipid degradation, but PPE's phenolic and vitamin content mitigated PUFA loss, maintaining nutritional quality (Kharchoufi et al. 2018). The high elaidic acid content in T4 (26.91% ± 0.77%, day 3) suggests a stable MUFA profile, though its reduction over time indicates oxidative susceptibility without sufficient antioxidant protection (Ebadi et al. 2019; Khodanazary 2019; Khodanazary et al. 2019; Rahul et al. 2024). These findings underscore the importance of PPE's antioxidant properties in preserving the nutritional and sensory quality of shrimp lipids, with potential applications in other high‐PUFA foods. Retention of PUFAs in coated samples is due to PPE's scavenging of free radicals, preventing chain reactions in lipid oxidation, which was more pronounced in controls as indicated by higher TBARS correlation (*r* = 0.92, *p* < 0.01) (Chen and Liu 2020).

**TABLE 14:** Saturated fatty acid (SFA) profile of shrimp treatments.

Days	Treatments	C14	C16	C18	C20	C22	C24
		Myristic acid	Palmitic acid	Stearic acid	Arachidic acid	Behenic acid	Lignoceric acid
The 3rd day	T1	3.20 ± 0.05^aA^	20.31 ± 0.83^aA^	5.84 ± 0.47^aA^	0.78 ± 0.08^abA^	0.65 ± 0.02^bA^	0.46 ± 0.05^bcA^
	T2	3.18 ± 0.04^aA^	19.91 ± 0.82^aA^	5.96 ± 0.47^aA^	0.99 ± 0.07^aA^	0.89 ± 0.02^aA^	0.53 ± 0.05^bA^
	T3	3.15 ± 0.04^aA^	20.38 ± 0.81^aA^	5.52 ± 0.46^aA^	0.45 ± 0.07^cC^	0.28 ± 0.01^cD^	0.36 ± 0.05^dB^
	T4	3.11 ± 0.04^aA^	20.91 ± 0.80^aA^	5.67 ± 0.46^aA^	0.47 ± 0.06^cD^	0.30 ± 0.00^cE^	0.37 ± 0.05^dC^
	T5	3.14 ± 0.05^aA^	20.38 ± 0.82^aA^	5.52 ± 0.46^aA^	0.45 ± 0.08^cA^	0.28 ± 0.02^cA^	0.36 ± 0.05^A^
	T6	3.23 ± 0.05^a^	20.91 ± 0.84^a^	5.67 ± 0.47^a^	0.47 ± 0.08^c^	0.30 ± 0.02^c^	0.37 ± 0.05^a^
The 6th day	T1	3.13 ± 0.04^aA^	20.29 ± 0.81^aA^	5.61 ± 0.46^aA^	0.55 ± 0.08^cB^	0.40 ± 0.02^cB^	0.39 ± 0.05^bB^
	T2	3.11 ± 0.04^aB^	20.10 ± 0.80^aA^	5.67 ± 0.46^aA^	0.66 ± 0.07^bB^	0.52 ± 0.02^cB^	0.42 ± 0.05^bB^
	T3	3.00 ± 0.04^aA^	19.30 ± 0.77^abA^	5.55 ± 0.44^aA^	0.74 ± 0.07^bA^	0.62 ± 0.01^bA^	0.44 ± 0.05^bA^
	T4	3.01 ± 0.04^aA^	19.22 ± 0.77^abA^	5.75 ± 0.45^aA^	0.95 ± 0.06^aA^	0.86 ± 0.00^aA^	0.51 ± 0.05^aA^
	T5	1.98 ± 0.03^bB^	12.84 ± 0.50^cB^	3.48 ± 0.29^bB^	0.28 ± 0.05^dB^	0.18 ± 0.01^dB^	0.22 ± 0.03^cB^
The 9th day	T1	2.82 ± 0.04^aAB^	18.26 ± 0.73^aB^	5.05 ± 0.41^aAB^	0.50 ± 0.07^cB^	0.36 ± 0.02^cC^	0.35 ± 0.05^bB^
	T2	2.96 ± 0.04^aB^	19.09 ± 0.77^aA^	5.38 ± 0.43^aAB^	0.62 ± 0.07^bB^	0.49 ± 0.01^bB^	0.40 ± 0.05^aB^
	T3	2.55 ± 0.04^aB^	16.40 ± 0.66^bB^	4.72 ± 0.36^aB^	0.63 ± 0.06^bB^	0.53 ± 0.01^bB^	0.37 ± 0.04^bB^
	T4	2.70 ± 0.04^aB^	17.30 ± 0.69^bB^	5.17 ± 0.40^aAB^	0.85 ± 0.05^aA^	0.77 ± 0.00^aB^	0.46 ± 0.05^aB^
The 12th day	T1	2.40 ± 0.03^aC^	15.52 ± 0.62^abC^	4.29 ± 0.35^aB^	0.42 ± 0.06^cC^	0.31 ± 0.01^dD^	0.30 ± 0.04^bBC^
	T2	2.66 ± 0.04^aC^	17.18 ± 0.69^aB^	4.84 ± 0.39^aB^	0.56 ± 0.06^bC^	0.44 ± 0.01^bC^	0.36 ± 0.04^aC^
	T3	1.91 ± 0.03^bC^	12.30 ± 0.49^cC^	3.54 ± 0.28^aC^	0.47 ± 0.04^cC^	0.39 ± 0.01^bcC^	0.28 ± 0.03^bC^
	T4	2.16 ± 0.03^aC^	13.84 ± 0.55^cC^	4.13 ± 0.32^aC^	0.68 ± 0.04^aB^	0.62 ± 0.00^aC^	0.38 ± 0.04^aC^
The 15th day	T1	2.04 ± 0.03^aD^	13.20 ± 0.53^bD^	3.65 ± 0.30^aC^	0.36 ± 0.05^bD^	0.26 ± 0.01^dE^	0.18 ± 0.03^bD^
	T2	2.40 ± 0.03^aD^	15.47 ± 0.62^aC^	4.36 ± 0.35^aC^	0.51 ± 0.05^aC^	0.40 ± 0.01^bD^	0.22 ± 0.04^aD^
	T3	1.53 ± 0.02^bD^	9.84 ± 0.39^cD^	2.83 ± 0.23^bD^	0.38 ± 0.03^bD^	0.32 ± 0.01^cD^	0.14 ± 0.03^cD^
	T4	1.84 ± 0.03^bD^	11.76 ± 0.47^bD^	3.15 ± 0.27^aD^	0.58 ± 0.04^aC^	0.53 ± 0.00^aD^	0.18 ± 0.03^bD^

*Note:* The letters a, b, … in each column indicate significant differences among treatments on the same day. The letters A, B, … indicate significant differences for each treatment over 15 days.

**TABLE 15:** Monounsaturated fatty acid (MUFAs) profile of shrimp treatments.

Days	Treatments	C14:1n5	C16:1n7	C18:1n9	C18:1n7	C20:1n9	C22:1n9	C24:1
		Myristoleic acid	Palmitoleic acid	Elaidic acid	Cis‐vaccenic acid	Gondoic acid	Erucic acid	Nervonic acid
The 3rd day	T1	0.17 ± 0.01^aA^	4.52 ± 0.02^aB^	26.19 ± 0.81^aA^	2.71 ± 0.12^aA^	2.67 ± 0.10^aA^	0.62 ± 0.07^aA^	0.29 ± 0.04^aA^
	T2	0.18 ± 0.01^aA^	4.47 ± 0.02^aA^	25.71 ± 0.80^aA^	2.69 ± 0.12^aA^	2.65 ± 0.10^aA^	0.63 ± 0.07^aA^	0.30 ± 0.04^aA^
	T3	0.15 ± 0.01^aA^	4.48 ± 0.02^aA^	26.23 ± 0.79^aA^	2.68 ± 0.12^aA^	2.63 ± 0.10^aA^	0.59 ± 0.07^aA^	0.28 ± 0.04^aA^
	T4	0.16 ± 0.01^aB^	4.61 ± 0.02^aA^	26.91 ± 0.77^aA^	2.75 ± 0.12^aA^	2.70 ± 0.10^aA^	0.61 ± 0.07^aA^	0.29 ± 0.04^aA^
	T5	0.15 ± 0.01^aA^	4.48 ± 0.02^aA^	26.23 ± 0.80^aA^	2.68 ± 0.12^aA^	2.63 ± 0.10^aA^	0.59 ± 0.07^aA^	0.28 ± 0.04^aA^
	T6	0.16 ± 0.01^a^	4.60 ± 0.02^a^	26.91 ± 0.82^a^	2.75 ± 0.12^a^	2.70 ± 0.10^a^	0.61 ± 0.08^a^	0.29 ± 0.04^a^
The 6th day	T1	0.15 ± 0.01^aB^	4.48 ± 0.02^aA^	26.13 ± 0.79^aA^	2.68 ± 0.12^aA^	2.63 ± 0.10^aA^	0.60 ± 0.07^aA^	0.28 ± 0.04^aA^
	T2	0.16 ± 0.01^aB^	4.45 ± 0.02^aA^	25.90 ± 0.79^aA^	2.67 ± 0.12^aA^	2.62 ± 0.0.10^aA^	0.60 ± 0.07^aA^	0.28 ± 0.04^aAB^
	T3	0.16 ± 0.01^aA^	4.29 ± 0.02^aB^	24.88 ± 0.75^abB^	2.58 ± 0.11^aB^	2.54 ± 0.09^aB^	0.59 ± 0.07^aA^	0.28 ± 0.04^aA^
	T4	0.18 ± 0.01^aA^	4.31 ± 0.02^aB^	24.81 ± 0.75^abB^	2.59 ± 0.11^aB^	2.56 ± 0.09^aB^	0.61 ± 0.07^aA^	0.29 ± 0.03^aA^
	T5	0.09 ± 0.01^bB^	2.82 ± 0.01^bB^	16.52 ± 0.50^cB^	1.69 ± 0.07^bB^	1.66 ± 0.06^bB^	0.37 ± 0.05^bB^	0.18 ± 0.02^bB^
The 9th day	T1	0.14 ± 0.01^aB^	4.03 ± 0.02^aC^	23.52 ± 0.72^abB^	2.41 ± 0.10^aB^	2.37 ± 0.10^aB^	0.54 ± 0.10^aB^	0.25 ± 0.04^aAB^
	T2	0.15 ± 0.01^aB^	4.23 ± 0.02^aB^	24.60 ± 0.75^aA^	2.54 ± 0.10^aB^	2.49 ± 0.10^aB^	0.57 ± 0.10^aA^	0.27 ± 0.04^aB^
	T3	0.14 ± 0.01^aAB^	3.65 ± 0.02^bC^	21.15 ± 0.64^bC^	2.19 ± 0.10^aC^	2.16 ± 0.10^aC^	0.50 ± 0.10^aB^	0.24 ± 0.03^aB^
	T4	0.16 ± 0.01^aB^	3.87 ± 0.02^bC^	22.33 ± 0.67^abC^	2.34 ± 0.10^aBC^	2.30 ± 0.10^aC^	0.55 ± 0.06^aB^	0.26 ± 0.03^aB^
The 12th day	T1	0.12 ± 0.01^bcC^	3.43 ± 0.01^aB^	19.99 ± 0.61^bC^	2.05 ± 0.09^aC^	2.01 ± 0.7^aC^	0.46 ± 0.06^aC^	0.22 ± 0.03^aAB^
	T2	0.14 ± 0.01^aB^	3.81 ± 0.02^aC^	22.14 ± 0.67^aB^	2.28 ± 0.10^aC^	2.24 ± 0.08^aC^	0.52 ± 0.06^aB^	0.24 ± 0.03^aC^
	T3	0.10 ± 0.01^dB^	2.74 ± 0.01^bD^	15.86 ± 0.48^dD^	1.64 ± 0.07^bD^	1.62 ± 0.06^bD^	0.38 ± 0.05^bC^	0.18 ± 0.02^cC^
	T4	0.13 ± 0.01^abC^	3.10 ± 0.01^aD^	17.87 ± 0.54^cD^	1.87 ± 0.08^bD^	1.84 ± 0.07^bD^	0.44 ± 0.05^aC^	0.21 ± 0.03^abC^
The 15th day	T1	0.10 ± 0.01^aD^	2.91 ± 0.01^abD^	16.99 ± 0.52^bD^	1.74 ± 0.08^bD^	1.71 ± 0.06^bD^	0.39 ± 0.05^abD^	0.18 ± 0.03^abC^
	T2	0.12 ± 0.01^aC^	3.43 ± 0.01^aD^	19.93 ± 0.60^aC^	2.05 ± 0.09^aD^	2.02 ± 0.07^aD^	0.46 ± 0.06^aC^	0.22 ± 0.03^aC^
	T3	0.08 ± 0.01^bC^	2.19 ± 0.01^cE^	12.69 ± 0.38^cE^	1.31 ± 0.06^bE^	1.29 ± 0.05^cE^	0.30 ± 0.04^cD^	0.14 ± 0.02^cD^
	T4	0.11 ± 0.01^aD^	2.63 ± 0.01^bE^	15.18 ± 0.46^bE^	1.59 ± 0.07^bE^	1.57 ± 0.06^bcE^	0.38 ± 0.04^abD^	0.18 ± 0.02^abD^

*Note:* The letters a, b, … in each column indicate significant differences among treatments on the same day. The letters A, B, … indicate significant differences for each treatment over 15 days.

**TABLE 16:** Polyunsaturated fatty acid (PUFAs) profile of shrimp treatments.

Days	Treatments	C18:2n6	C18:3	C20:2n6	C20:3n3	C20:4n6	C20:5n3	C22:6n3
		Linoleic acid	Linolenic acid	Eicosadienoic acid (EDA)	Eicosatrienoic acid	Arachidonic acid	EPA	DHA
The 3rd day	T1	7.32 ± 0.11^aA^	1.74 ± 0.05^aA^	0.38 ± 0.11^bA^	0.31 ± 0.04^aA^	0.75 ± 0.08^aA^	5.04 ± 0.82^aA^	11.25 ± 0.89^aA^
	T2	7.19 ± 0.11^aA^	1.78 ± 0.05^aA^	0.49 ± 0.11^aA^	0.33 ± 0.04^aA^	0.77 ± 0.08^aA^	5.00 ± 0.81^aA^	11.38 ± 0.89^aA^
	T3	7.34 ± 0.11^aA^	1.64 ± 0.05^aA^	0.20 ± 0.11^cC^	0.27 ± 0.04^aA^	0.70 ± 0.08^aA^	4.97 ± 0.80^aA^	10.79 ± 0.88^aA^
	T4	7.53 ± 0.11^aA^	1.69 ± 0.05^aA^	0.21 ± 0.11^cD^	0.28 ± 0.04^aA^	0.73 ± 0.08^aA^	5.10 ± 0.78^aA^	11.07 ± 0.088^aA^
	T5	7.35 ± 0.11^aA^	1.64 ± 0.05^aA^	0.20 ± 0.11^cA^	0.27 ± 0.04^aA^	0.70 ± 0.07^aA^	4.97 ± 0.80^aA^	10.79 ± 0.87^aA^
	T6	7.53 ± 0.11^a^	1.69 ± 0.05^a^	0.21 ± 0.11^c^	0.28 ± 0.04^a^	0.73 ± 0.08^a^	5.10 ± 0.83^a^	11.07 ± 0.89^a^
The 6th day	T1	7.31 ± 0.11^aA^	1.67 ± 0.05^aB^	0.25 ± 0.02^bB^	0.28 ± 0.04^aB^	0.72 ± 0.08^aA^	4.98 ± 0.80^aA^	10.90 ± 0.87^aB^
	T2	7.24 ± 0.10^aA^	1.69 ± 0.05^aB^	0.31 ± 0.03^abB^	0.29 ± 0.04^aB^	0.73 ± 0.08^aA^	4.96 ± 0.79^aA^	10.97 ± 0.87^aB^
	T3	6.96 ± 0.10^abB^	1.65 ± 0.05^aA^	0.36 ± 0.03^abA^	0.29 ± 0.04^aA^	0.71 ± 0.07^aA^	4.79 ± 0.76^aA^	10.69 ± 0.84^aA^
	T4	6.94 ± 0.10^abB^	1.72 ± 0.05^aA^	0.48 ± 0.03^aA^	0.32 ± 0.04^aA^	0.75 ± 0.07^aA^	4.82 ± 0.75^aB^	10.98 ± 0.84^aA^
	T5	4.62 ± 0.07^cB^	1.03 ± 0.03^bB^	0.12 ± 0.01^cB^	0.17 ± 0.02^bB^	0.44 ± 0.05^bB^	3.13 ± 0.51^bB^	6.80 ± 0.55^bB^
The 9th day	T1	6.58 ± 0.10^aB^	1.50 ± 0.04^aC^	0.23 ± 0.02^bB^	0.25 ± 0.04^bBC^	0.64 ± 0.07^abB^	4.48 ± 0.72^aB^	9.81 ± 0.79^aC^
	T2	6.88 ± 0.10^aB^	1.60 ± 0.04^aB^	0.30 ± 0.02^abB^	0.28 ± 0.04^aB^	0.69 ± 0.07^aB^	4.71 ± 0.75^aB^	10.42 ± 0.83^bB^
	T3	5.91 ± 0.10^abC^	1.41 ± 0.04^aB^	0.30 ± 0.02^abB^	0.25 ± 0.03^bA^	0.61 ± 0.06^bB^	4.07 ± 0.64^bB^	9.09 ± 0.71^aAB^
	T4	6.24 ± 0.10^aC^	1.55 ± 0.04^abB^	0.43 ± 0.03^aA^	0.29 ± 0.03^aA^	0.67 ± 0.06^aB^	4.35 ± 0.68^abC^	9.88 ± 0.76^aB^
The 12th day	T1	5.59 ± 0.10^abC^	1.27 ± 0.04^abD^	0.19 ± 0.02^cBC^	0.21 ± 0.03^bDE^	0.55 ± 0.10^abC^	3.81 ± 0.61^bC^	8.34 ± 0.69^aD^
	T2	6.19 ± 0.10^aC^	1.44 ± 0.04^aC^	0.27 ± 0.02^abBC^	0.25 ± 0.03^aC^	0.62 ± 0.10^aB^	4.24 ± 0.68^aC^	9.38 ± 0.74^aBC^
	T3	4.43 ± 0.10^abD^	1.05 ± 0.03^bC^	0.23 ± 0.02^bBC^	0.19 ± 0.02^bB^	0.45 ± 0.05^cC^	3.05 ± 0.48^cC^	6.81 ± 0.54^bC^
	T4	4.99 ± 0.10^abD^	1.24 ± 0.03^abC^	0.34 ± 0.02^aB^	0.23 ± 0.03^abB^	0.54 ± 0.05^abC^	3.48 ± 0.54^bD^	7.91 ± 0.61^abC^
The 15th day	T1	4.75 ± 0.07^bD^	1.08 ± 0.03^bE^	0.16 ± 0.02^cC^	0.18 ± 0.03^abE^	0.47 ± 0.05^bD^	3.24 ± 0.52^bD^	7.09 ± 0.57^aE^
	T2	5.57 ± 0.08^aD^	1.30 ± 0.04^aD^	0.24 ± 0.02^bC^	0.23 ± 0.03^aCD^	0.56 ± 0.06^aC^	3.81 ± 0.61^aD^	8.44 ± 0.67^aC^
	T3	3.55 ± 0.05^cE^	0.84 ± 0.02^cD^	0.18 ± 0.01^cC^	0.15 ± 0.02^cC^	0.36 ± 0.04^cD^	2.44 ± 0.39^dD^	5.45 ± 0.43^bcC^
	T4	4.24 ± 0.06^bE^	1.05 ± 0.03^bD^	0.29 ± 0.02^aBC^	0.19 ± 0.02^abC^	0.46 ± 0.04^bD^	2.96 ± 0.46^cE^	6.72 ± 0.52^bC^

*Note:* The letters a, b, … in each column indicate significant differences among treatments on the same day. The letters A, B, … indicate significant differences for each treatment over 15 days.

## 4 Conclusion

Pomegranate peel extract (PPE), rich in bioactive compounds such as catechins, ellagic acid, and omega‐3 fatty acids, emerges as a potent natural preservative with robust antibacterial and antioxidant properties. This study demonstrated that alginate‐gum arabic coatings enriched with 0.5% (T1) and 1% (T2) encapsulated PPE significantly enhanced the physicochemical, microbial, and sensory attributes of shrimp ( *Litopenaeus vannamei* ) during 15 days of refrigerated storage at 4°C. Specifically, the thiobarbituric acid reactive substances (TBARS) index, indicative of lipid oxidation, was lowest in T2 (0.18 ± 0.01 mg MDA/kg) on day 3, reflecting superior oxidative stability. Texture parameters, including firmness (T5: 4.75 ± 0.06 g) and resilience (T1: 1.18 ± 0.07), peaked on day 3, while sensory scores for taste, texture, color, odor, and overall acceptance were significantly higher (*p* < 0.05) in T1 and T2 compared to other treatments. Total bacterial counts (TBC) were lowest in T1 (5.50 ± 0.57 log CFU/g) and T2 (5.00 ± 0.00 log CFU/g) on day 3, maintaining significantly lower levels (*p* < 0.05) throughout storage, underscoring PPE's antimicrobial efficacy. The novelty of this study lies in the innovative encapsulation of PPE within an alginate‐gum arabic biopolymer matrix, which facilitates controlled release of bioactive compounds, enhancing their stability and efficacy compared to traditional non‐encapsulated or chitosan‐based coatings (Ebadi et al. 2019; Khodanazary 2019; Khodanazary et al. 2019; Rahul et al. 2024). This approach leverages the synergistic film‐forming properties of alginate and gum arabic, providing a sustainable, biodegradable coating that valorises pomegranate peel waste, aligning with circular economy principles (Siddiqui et al. 2024). The high phenolic content (e.g., catechins: 896.91 ± 26.26 mg/100 g) and preservation of polyunsaturated fatty acids (PUFAs), such as DHA (11.38% ± 0.89% in T2, day 3) and EPA (5.10% ± 0.78% in T4, day 3), highlight PPE's potential as a natural alternative to synthetic preservatives, offering both functional and nutritional benefits for seafood preservation. These findings position PPE‐enriched coatings as a promising solution for extending the shelf life of perishable seafood while maintaining quality and safety. However, future research should focus on optimizing encapsulation techniques to further enhance bioactive release kinetics and explore the scalability and cost‐effectiveness of PPE‐based coatings for industrial applications. Additionally, investigations into the sensory impact of higher PPE concentrations and the long‐term stability of encapsulated coatings under varying storage conditions could further refine their commercial viability (Mo et al. 2022). Such advancements could facilitate the adoption of PPE‐based coatings in the food industry, contributing to sustainable preservation strategies and reducing reliance on synthetic additives.

## Author Contributions

**Fatemeh Rahmati:** investigation (equal). **Sara Jafarian:** supervision (equal). **Peiman Ariaii:** supervision (equal), validation (equal). **Mahdi Sharifi Soltani:** methodology (equal). **Leila Roozbeh Nasiraei:** methodology (equal).

## Funding

The authors have nothing to report.

## Ethics Statement

This study did not involve human or animal subjects requiring ethical approval. All experiments were conducted in accordance with standard laboratory practices and ethical guidelines.

## Conflicts of Interest

The authors declare no conflicts of interest.

## Data Availability

The data supporting the findings of this study are available from the corresponding author upon reasonable request.
